# Effects of loss aversion on post-decision wagering: Implications for measures of awareness

**DOI:** 10.1016/j.concog.2009.11.002

**Published:** 2010-03

**Authors:** Stephen M. Fleming, Raymond J. Dolan

**Affiliations:** Wellcome Trust Centre for Neuroimaging, University College London, 12 Queen Square, London WC1N 3BG, United Kingdom

**Keywords:** Post-decision wagering, Perception, Economics, Signal detection theory, Reward, Awareness, Metacognition, Psychophysics

## Abstract

Wagering contingent on a previous decision, or post-decision wagering, has recently been proposed to measure conscious awareness. Whilst intuitively appealing, it remains unclear whether economic context interacts with subjective confidence and how such interactions might impact on the measurement of awareness. Here we propose a signal detection model which predicts that advantageous wagers placed on the identity of preceding stimuli are affected by loss aversion, despite stimulus visibility remaining constant. This pattern of predicted results was evident in a psychophysical task where we independently manipulated perceptual and economic factors. Changes in wagering behaviour induced by changes in wager size were largely driven by changes in criterion, consistent with the model. However, for near-threshold stimuli, a reduction in wagering efficiency was also evident, consistent with an apparent but potentially illusory decrease in awareness of the stimulus. These findings challenge an assertion that post-decision wagering provides a direct index of subjective awareness.

## 1 Introduction

Knowledge of one’s own uncertainty regarding an outcome plays a key role in determining decision strategy (Foote & Crystal, 2007; Hampton, 2001; Kepecs, Uchida, Zariwala, & Mainen, 2008; Kiani & Shadlen, 2009; Smith, Shields, & Washburn, 2003), and may even be a central property of awareness (Cleeremans, Timmermans, & Pasquali, 2007; Dienes, 2008; Rosenthal, 2000, 2002). For example, knowing in advance that you are unlikely to pass a test makes you reluctant to take the test in the first place (Higham, 2007; Higham & Gerrard, 2005; Koriat & Goldsmith, 1996; Metcalfe & Finn, 2008), and may reflect awareness of your own (low) ability.

Such insights motivated the use of wagering contingent on decision performance (post-decision wagering) as a direct and intuitive measure of conscious awareness (Koch & Preuschoff, 2007; Persaud & McLeod, 2008; Persaud, McLeod, & Cowey, 2007). In its simplest form, a participant is required to detect whether an arbitrary stimulus is present or not and then, in a second stage, gamble on whether his/her response was correct or not. Advantageous wagers are made when participants accurately assess the level of sensory evidence on each trial, linking objective performance to the subjectivity of stimulus information (Persaud et al., 2007). Furthermore, using wagers to measure awareness can potentially overcome problems of interpretation associated with a lack of control over subjective reports (Eriksen, 1960) and the motivation of a subject (cf. Visser & Merikle, 1999). However, the interpretation of post-decision wagering data is potentially confounded by complex factors affecting how gains and losses affect performance (Clifford, Arabzadeh, & Harris, 2008; Dienes & Seth, 2009; Schurger & Sher, 2008), and a full behavioural analysis of advantages, and disadvantages, in defining consciousness based on these economic terms is lacking.

**Fig. 1:**
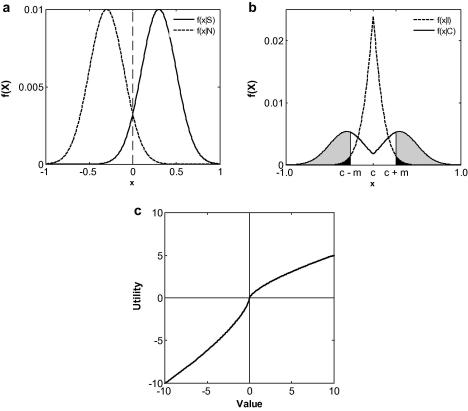
Computational model of post-decision wagering performance. (a) Theoretical distributions over a random variable *X* (corresponding to an arbitrary stimulus axis) for signal (*S*, solid line) and noise (*N*, broken line). (b) Probability distributions over different values of *X* for the probability of making a correct (solid line) and incorrect (broken line) categorisation. Shaded areas represent the integrals specified in Eqs. (9) (*H*_w_, grey) and (10) (*FA*_w_, black). (c) Schematic of the loss-averse utility function used in the model.

The processing stages underlying post-decision wagering can be logically decomposed using signal detection theory (SDT). In the first instance, it is assumed that objective features of the stimulus lead to a perceptual effect, modelled as a univariate random variable *X* (Fig. 1a), which we label “sensory evidence”. Discrimination errors at this stage are inevitable if the difference in sensory evidence between signal and noise (or other arbitrary category discriminations) is weak, leading to overlapping probability distributions (Fig. 1a). It is thus assumed that participants make a decision by splicing up an evidence axis into two halves using a decision criterion (Green & Swets, 1966). In a post-decision wagering experiment, the participant is now required to make a high or low wager on whether they were correct. This requires knowledge both of how likely it was that the first decision was correct, and their expected return from the wager (Clifford et al., 2008). The former can be recovered from the sensory evidence on a given trial (Galvin, Podd, Drga, & Whitmore, 2003). The latter, however, is psychologically more complex given evidence that people are loss averse (Kahneman & Tversky, 1979; Tversky & Kahneman, 1991), manifesting in a propensity to weight losses greater than gains when making value-based decisions. Loss aversion has been suggested to impact on post-decision wagering performance by modulating a link between the assessment of sensory evidence and overt confidence (Schurger & Sher, 2008).

To quantify how wagering on sensory evidence is influenced by economic factors, we first developed a simple computational model of post-decision wagering behaviour, building on previous work by Galvin and colleagues (Galvin et al., 2003), but now extending this framework to encompass loss aversion. Using this model, we derive precise predictions about how sensory evidence translates into advantageous wagering responses. The accuracy of metacognitive assessments can be intuited as how transparent the initial decision process is to a putative “higher” level assessment. For example, if there is ambiguity in the decision process then the categorisation of one’s own performance as being correct, or incorrect, will be subject to error. This intuition can be captured within the logic of signal detection theory (SDT), which assesses how faithfully an organism separates signal from noise (Green & Swets, 1966; Macmillan & Creelman, 2005). In standard applications of SDT (Type I), detection performance is assessed by a comparison of the proportion of “hits” and “false alarms” in a stimulus detection task. By applying the logic of SDT to post-decision wagering, we categorise a “hit” as a high wager after a correct decision and a “false alarm” as a high wager after an incorrect decision (see Table 1). This type of analysis is known as Type II SDT (Clarke, Birdsall, & Tanner, 1959; Clifford et al., 2008; Galvin et al., 2003; Kunimoto, Miller, & Pashler, 2001; Macmillan & Creelman, 2005). On the basis of these considerations, we derive a theoretical relationship linking Type I and Type II task performance by applying an ideal observer framework, enabling us to explore the relationship between post-decision wagering and changes in sensory evidence. Our specific prediction here was that post-decision wagering would be influenced by loss aversion, decoupling a link between perception and confident responding. To test our model, we examine data from a psychophysical experiment in which subjects made a high or low wager following a simple sensory judgement.

## 2 Methods

### 2.1 Signal detection model

In signal detection theory (SDT), a Type I decision is based upon overlapping Gaussian probability distributions over a random variable *X*, conditional on the events signal (*S*) and noise (*N*) (Fig. 1a). Assuming an unbiased response criterion, *c*, for the Type I detection decision (Galvin et al., 2003), we can specify the distribution over *X* for the probability of the Type I response being correct or incorrect:

(1) $$f(x|C)=\left\{\begin{array}{cc} \frac{f(x|N)}{p(C)}\text{,} & x⩽c\\ \frac{f(x|S)}{p(C)}\text{,} & x>c \end{array}\right)$$

(2) $$f(x|I)=\left\{\begin{array}{cc} \frac{f(x|S)}{p(I)}\text{,} & x⩽c\\ \frac{f(x|N)}{p(I)}\text{,} & x>c \end{array}\right)$$

where *p*(*C*) and *p*(*I*) are the average probabilities of making a correct or incorrect response on any given trial (Macmillan & Creelman, 2005). Full derivations of Eqs. (1) and (2) can be found in (Galvin et al., 2003); the (constant) prior terms from their more general analysis are omitted here for clarity. The distributions specified by (1) and (2) are plotted graphically in Fig. 1b. It is important to note that these Type II distributions are conditional transformations based on whether the first decision was correct or not. That is, the shape of the *f*(*x*|*C*) curve follows the signal distribution when *x* > *c* (a Type I hit) and the noise distribution when *x* < *c* (a Type I correct rejection). Similarly, the shape of the *f*(*x*|*I*) curve follows shape of the noise distribution when *x* > *c* (a Type I false alarm) and the signal distribution when *x* < *c* (a Type I miss). The heights of both *f*(*x*|*C*) and *f*(*x*|*I*) are then scaled so that they sum to one.

A correct Type II response is more likely towards the left or right-hand extremes of *X* in Fig. 1b (high signal or high noise trials), whereas incorrect responses predominate where there is maximal overlap between signal and noise. The inherent assumption here is that the uncertainty associated with being sure of seeing something is the same as the uncertainty associated with being sure of not seeing something (the Type II distributions are symmetric around *X* = 0), an assumption we return to under Sections 3 and 4.

The log-likelihood of being correct on any given trial (likelihood ratio; *LR*) is the log of the ratio of (1) and (2):

(3) $$\mathrm{LR}=\log\left(\frac{f(x|C)}{f(x|I)}\right)$$

We assume that high wagers are made when the log-likelihood of being correct on the Type I task reaches a given criterion, *β_w_*. As the likelihood ratio is symmetric around *c*, there are thus two values of *x* for each *β_w_*, one for when *x* ⩽ *c* and one for when *x* > *c*. This corresponds to being sure that a signal was or was not present and wagering high.

In standard applications of SDT, the optimal placement of the log-likelihood ratio criterion can be calculated based on the relative utility of making a hit (*H*), miss (*M*), correct rejection (*CR*) or false alarm (*FA*) (assuming equal priors), as follows (Macmillan & Creelman, 2005):

(4) $$\beta =\log\left(\frac{R(\mathrm{CR})-R(\mathrm{FA})}{R(H)-R(M)}\right)$$

In Type I SDT, it is likely that the “reward” (R) for a false alarm or miss will be negative. Imagine taking part in a psychophysical task where the penalty was £1 for a false alarm and 1p for a miss, with 10p earned for correct responses. The optimal value of *β* would then be (0.1 − (−1))/(0.1 − (−0.01)) = 10: the odds favouring stimulus *B* should be 10:1 or better in order to risk a “*B*” response (that associated with the false alarm penalty). In our Type II analysis, we similarly assume that subjects base their response criterion, *β_w_*, on the relative utility of the four possible Type II wagering outcomes, which are mapped onto the four SDT categories of Eq. (4) using Table 1. This ratio is then adjusted by the prior probability of the trial being correct or incorrect (*P*(*C*) and *P*(*I*)):

(5) $$\beta_{w}=\log\left(\frac{C_{L}-C_{H}}{R_{H}-R_{L}}.\frac{P(I)}{P(C)}\right)$$

In contrast to Eq. (4) for Type I SDT, in Eq. (5) both hits and misses are associated with positive outcomes (trials are labelled “misses” when the Type I response was correct but only the low wager was used, leading to a lower reward than could have been obtained, but still a reward; cf. Kunimoto et al., 2001). Thus, the terms in the denominator of Eq. (5) are the positive utilities of each wager (*R*), whereas the outcomes in the numerator are associated with the negative costs of losing each wager (*C*) following incorrect responses.

Crucially, we incorporate asymmetric utility curves when calculating the values of *R* and *C*, as proposed within Prospect Theory (Kahneman & Tversky, 1979). A schematic of such a curve is shown in Fig. 1c. Risk aversion for mixed (gain or loss) gambles is explained through loss aversion, such that given the same absolute value losses carry a greater impact than gains (Tversky & Kahneman, 1991). This nonlinear relationship between gain (*R*) and loss (*C*) leads to a value of *β_w_* that is dependent on the size of the high wager, assuming the low wager remains fixed for simplicity.

The positive and negative utility of each wager is specified as follows:

(6) $$R=V^{r}\text{,}\;V>0$$

(7) $$C=s{\left(-V\right)}^{t}\text{,}\;V<0$$

where *V* is the objective value of the wager, *r* and *t* are power functions for the gain and loss domain respectively, and *s* is the loss aversion index. We restricted *r* and *t* such that 0 < *r* < *t* < 1, resulting in utility being concave for gains, convex for losses and more linear in losses than in gains (Fennema & Van Assen, 1999; Kobberling & Wakker, 2005). We define *c* ± *m* (as the likelihood ratio is symmetric about *c*) as values of *x* such that:

(8) $$\log\left(\frac{f(x|C)}{f(x|I)}\right)=\beta_{w}$$

Using the signal detection categories of Table 1, we then compute theoretical hit and false alarm rates for a range of values of *m* by integrating over the Type II probability distributions specified in (1), (2) and Fig. 1b:

(9) $$H_{w}=\int_{-\infty }^{c-m}f(x|C)\mathrm{dx}+\int_{c+m}^{\infty }f(x|C)\mathrm{dx}$$

(10) $${\mathrm{FA}}_{w}=\int_{-\infty }^{c-m}f(x|I)\mathrm{dx}+\int_{c+m}^{\infty }f(x|I)\mathrm{dx}$$

### 2.2 Psychophysics

**Fig. 2:**
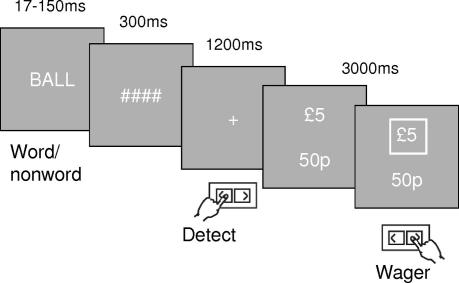
Psychophysical task design. Participants were required to detect whether a masked stimulus was a word or a non-word, and then place a high or low wager on whether their initial response was correct or not. To increase stimulus uncertainty, the target and mask could appear at any one of four locations around a central fixation cross (not shown).

Thirteen participants (3 male, 10 female, mean age 27 ± 8.6 years) performed a lexical decision task spread over two sessions on consecutive days, each contributing 1440 trials to the analysis. The task was to decide whether a heavily masked stimulus was a word or a non-word, and then to gamble either “high” or “low” depending on the correctness of their initial judgement (Fig. 2). One-hundred and seventy-two four-letter words were taken from the MRC Psycholinguistic Database (Wilson, 1988), and matched for familiarity and written frequency of occurrence. A related set of pronounceable non-words was created by a random vowel change. To increase task difficulty, stimuli appeared randomly at any one of four locations surrounding a central fixation point. Threshold detection performance (79%) was assessed for each individual using a staircase procedure (Levitt, 1971); on each trial stimulus duration was 100%, 80% or 50% of this threshold. The low wager remained constant across blocks at 50p; the value of the high wager varied across blocks (£1, £2, £5 or £10). Responses were made using the left-hand (“*z*” or “*x*” keys) to indicate the word/non-word, and the right-hand (“up” or “down” arrow keys) to indicate a high or low gamble.

Stimulus duration was randomised within blocks, giving a 3 × 4 factorial design crossing high wager size (£1, £2, £5 or £10) with stimulus visibility (100%, 80% or 50% of threshold duration). This design allowed us to recover an estimate of advantageous wagering (proportion of high wagers following correct responses; “hits” in Table 1) for each cell in our design.

Participants were informed that the wager they were making was a true mixed gamble: they would win the money they wagered if their initial word/non-word decision was correct, and would have this amount deducted from their running total if they were incorrect. They were also informed that only one trial from each block was to be evaluated at random, allowing us to use relatively larger amounts of money while still encouraging participants to treat each trial as a meaningful gamble.

### 2.3 Analysis

To quantify a match between the psychophysical data and the model, we computed the likelihood of each subject’s wagers under the model (from the integrals in Eqs. (9) and (10)), optimised over free parameters of the utility function. We compared two parameterisations of the utility function: one with, and one without, a loss aversion constant (Tversky & Kahneman, 1991). We took the parameters *r*, *t* and *s* to be free in Model 1 (loss averse + power), and *r* and *t* to be free in Model 2 (power). The testable model space was constrained by requiring the loss and gain domains of the utility function to be asymmetric, as symmetric functions do not predict any effect of wager size on the Type II response criterion (see Supplementary Material for a more detailed discussion of empirical forms of the utility curve).

Type I sensitivity was set for each level of stimulus visibility using empirically estimated *d*′ values from each subject. For each model, we fit the free parameters to each participant’s choice data by maximising the likelihood of the observed choices, using a nonlinear optimisation algorithm (*fminsearch* in the Matlab Optimisation toolbox). We report negative log-likelihoods (smaller values indicate better fit), penalised for model complexity using the Bayesian information criterion (BIC) (Schwarz, 1978). To compare models at the group level we summed each subject’s BIC, resulting in a log group Bayes factor (Stephan, Weiskopf, Drysdale, Robinson, & Friston, 2007).

For signal detection analysis, Type I “word” responses were classified as hits or false alarms; “non-word” responses were classified as correct rejections or misses. For both the model and the data we computed Type II hit and false alarm rates using the classification scheme of Table 1. To allow comparison with previous datasets employing post-decision wagering (Persaud & McLeod, 2008; Persaud et al., 2007), advantageous wagers were defined as Type II hit rates (proportion of high gambles made after correct decisions). A repeated-measures analysis of variance (ANOVA) was used to analyse these proportions, following an arcsine transformation of the data to correct for heterogeneity of variance. In addition Type I SDT measures of the initial perceptual discrimination (*d*′ and *c*) and Type II sensitivity ($d_{w}^{\prime }$) and criterion (*c_w_*) for the wagering response were calculated as follows (where *z* is the inverse of the cumulative normal distribution function):

(11) $$d^{\prime }=z(H)-z(F)$$

(12) $$d_{w}^{\prime }=z(H_{w})-z(F_{w})$$

(13) $$c=-0.5\left[z(H)+z(F)\right]$$

(14) $$c_{w}=-0.5\left[z(H_{w})+z(F_{w})\right]$$

0.5 was added to all cells in calculation of both Type I and Type II SDT measures to avoid extreme proportions of *H* or *F* (Hautus, 1995). SDT measures calculated for each wager and sensitivity condition were analysed using repeated-measures ANOVAs.

## 3 Results

### 3.1 Model comparison

**Fig. 3:**
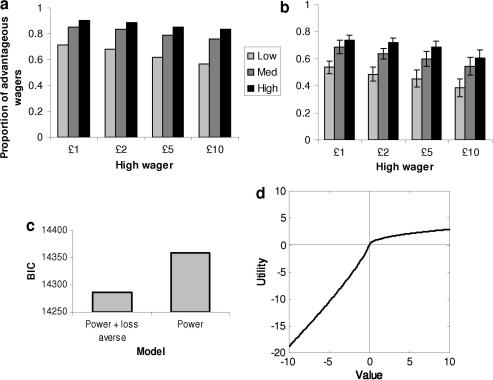
Comparison of computational model with psychophysical data. (a) Predicted proportions of advantageous wagers (proportion of high wagers following correct initial responses) derived from the computational model illustrated in Fig. 1, plotted as a function of stimulus visibility (low, medium and high) and high wager size. Stimulus visibility in the model was set to be equal to the empirically derived mean *d*′ values from the psychophysical task. (b) Mean (±SEM) observed proportions of advantageous wagers from 13 subjects in the word/non-word detection task. (c) Negative log-likelihoods of the model fits to the psychophysical data summed over subjects, penalised for model complexity using Bayesian information criterion (BIC). More negative values indicate a better fit, with a difference of three indicating strong evidence for one model compared to the other (Penny, Stephan, Mechelli, & Friston, 2004). It can be seen that despite the penalty for an extra parameter, the model with the loss aversion constant *s* provides a better fit to the data. (d) Utility function created by averaging the best-fit parameters from the power + loss averse model over subjects.

As an initial examination of the validity of our model, we computed estimates of advantageous wagering (the proportion of high wagers following correct responses) from both the model output and psychophysical data (Fig. 3a and b). Perceptual sensitivity levels in the model were set to match mean *d*′ values across subjects in the psychophysical experiment. It is clear that advantageous wagering is predicted not only to increase as a function of perceptual sensitivity, but also to decrease as a function of wager size. The latter decrease is a direct consequence of the loss-averse utility function implemented in the model.

We next tested the model predictions against psychophysical data. Advantageous wagering decreased with both decreasing stimulus visibility (effect of visibility; *F*_(2,24)_ = 99.87, *p* < .0001) and with increasing wager size (effect of wager; *F*_(3,36)_ = 10.71, *p *< .0001; Fig. 3b), closely matching the predictions of the model (Fig. 3a). Even when the stimulus was most visible, the size of the wager significantly modulated a tendency to gamble high following correct responses (paired *t*-test between £1 and £10 wagers; *t*_(12)_ = 3.90, *p *= .002). Indeed, for the range of stimulus durations we tested here, the effect of wager size did not interact with stimulus visibility (*F*_(6,72)_ < 1, *p *> .5).

In assessing the match between the psychophysical results and the model, we compared two parameterisations of the utility function: one with a loss aversion constant (Tversky & Kahneman, 1991) and one without. The model with the loss aversion constant was strongly preferred, despite the Bayesian information criterion (BIC) measure including a penalty for additional complexity (Fig. 3c; BIC difference of 73.2, corresponding to an *e*^73.2^ difference in Bayes factor). This suggests the transformation of perceptual sensitivity into confident wagering was indeed significantly affected by the loss aversion parameter of the model. The average utility function adopted by participants is shown in Fig. 3d. Across subjects, the loss aversion parameter *s* ranged from 1.94 to 4.53, with a mean of 2.74 ± 0.92. Such a parameter range is comparable with previous findings in the literature (Tom, Fox, Trepel, & Poldrack, 2007; Tversky & Kahneman, 1991).

### 3.2 Type I signal detection analysis

**Fig. 4:**
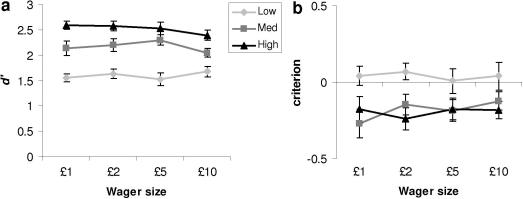
Word/non-word task performance. Subjects’ performance (*d*′) (a) and criterion (b) in the Type I detection task as a function of both visibility and wager size. Error bars reflect standard errors of the mean.

As expected, longer stimulus durations were associated with higher Type I *d*′ (*F*_(2,24)_ = 123.47, *p *< .0001). Importantly, however, Type I perceptual sensitivity remained constant across changes in wager size (interaction of wager size and visibility: *F*_(6,72)_ = 1.07, *p *> .3; Fig. 4a). As outlined in Section 2, an explicit assumption of our model is that Type I response criteria are unbiased. To evaluate this assumption we calculated Type I criterion for the word/non-word discrimination as a function of both stimulus visibility and wager size (Fig. 4b). A consistent bias towards responding “word” for the higher visibility trials was observed, leading to a significant effect of visibility on response criterion (*F*_(2,24)_ = 23.48, *p *< .0001). However, as for *d*′, this bias was expressed consistently across changes in wager size (no interaction with wager size; *F*_(6,72)_ = 1.31, *p *> .2), suggesting that these criterion shifts are not sufficient to explain biases in post-decision wagering induced by loss aversion.

### 3.3 Type II signal detection analysis

The results described under Model Comparison above show that a typical post-decision wagering measure – Type II hit rate – is subject to a bias that can be explained by loss aversion, despite the underlying Type I sensitivity to the stimulus remaining constant. However, participants’ wagering efficiency (ability to discriminate between correct and incorrect trials) might still be independent of their overall propensity to wager high (Higham, 2007; Kunimoto et al., 2001; but see Evans & Azzopardi, 2007). In other words, despite using the £10 wager less than the £1 wager for a given level of sensitivity (Fig. 3b), does this usage still discriminate well between correct and incorrect decisions?

**Fig. 5:**
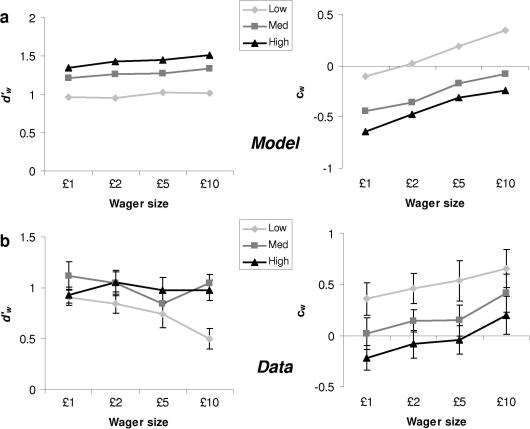
Type II signal detection analysis of wagering responses. (a) Predictions from the model output for the pattern of signal detection parameters ($d_{w}^{\prime }$, left-hand panel; *c_w_*, right-hand panel) calculated using Table 1. Wagering efficiency ($d_{w}^{\prime }$) is expected to change as a function of Type I *d*′, but only slightly as a function of wager size; the wagering criterion (*c_w_*) is expected to be affected by both wager size and Type I *d*′. (b) Type II signal detection parameters from the post-decision wagering task as a function of stimulus visibility and wager size. Error bars reflect standard errors of the mean.

To answer this question we computed Type II measures of wagering efficiency, $d_{w}^{\prime }$, and wagering criterion, *c_w_* (see Section 2) from both model and psychophysics data, as a function of stimulus visibility and wager size (Fig. 5a and b). The model predicts that wager size should primarily affect *c_w_* (due to loss aversion), without affecting $d_{w}^{\prime }$, which is a function of Type I *d*′ (Fig. 5a; left-hand panel). The data indeed showed a strong main effect of wager size on *c_w_* (*F*_(2,24)_ = 45.87, *p *< .0001), which did not interact with stimulus visibility (*F*_(6,72)_ = 0.56, *p *> .7), in agreement with the model (right-hand panel of Fig. 5b). The model also predicts a main effect of stimulus visibility on *c_w_*, in that wagering is generally more conservative when the task is more difficult (Fig. 5a, right-hand panel), an effect borne out in the data (*F*_(3,36)_ = 8.93, *p *< .005).

**Fig. 6:**
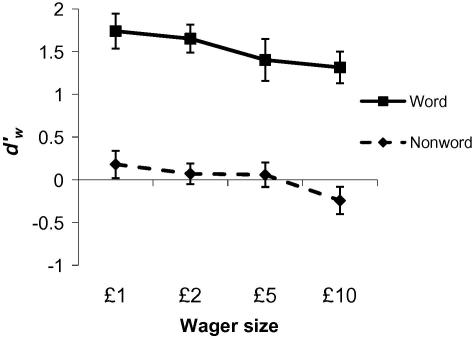
Type II sensitivity ($d_{w}^{\prime }$) for near-threshold stimuli as a function of wager size and stimulus type (word/non-word). A significant effect of wager size on $d_{w}^{\prime }$ was found that did not interact with stimulus type. Monitoring of performance following responses to words (targets) was increased compared to monitoring of responses to non-words (distractors). Error bars reflect standard errors of the mean.

The match between the model and the data on $d_{w}^{\prime }$ was less consistent. As predicted, we found a main effect of stimulus visibility (*F*_(2,24)_ = 7.78, *p *< .005; Fig. 5a), which did not show any significant interactions with wager size (*F*_(6,72)_ = 1.78, *p *> .1). However, for the lowest visibility, we noted a downward trend in $d_{w}^{\prime }$ as wager size increased, reflected in a linear effect of wager size that interacted with visibility level (*F*_(1,12)_ = 8.66, *p *= .012). Indeed, when comparing participants’ sensitivity to their own correctness in low-visibility judgements for £1 and £10 wager blocks, $d_{w}^{\prime }$ dropped by almost half (from 0.91 to 0.50). To ensure that these effects were not stimulus-specific (cf. Higham, Perfect, & Bruno, 2009; Experiment 3), we reanalysed the low-visibility condition as a function of whether the stimulus was a word or non-word (Fig. 6). A main effect of wager size on $d_{w}^{\prime }$ was confirmed (*F*_(3,36)_ = 3.95, *p *= .016) which did not interact with stimulus type (*F*_(3,36)_ < 1). A main effect of stimulus type was also found (*F*_(1,12)_ = 34.4, *p *< .001), replicating Higham et al.’s (2009) findings that monitoring is worse for distractors (non-words in our task) than targets (words). Overall, our results show that the size of the wager largely affects the criterion for using the high-wager response, but for near-threshold stimuli, also affects metacognitive performance.

### 3.4 Test of model assumptions

An inherent assumption of our model is that the wagering criterion (*m* in Fig. 1b) is symmetric about *c*. In other words, it is assumed that the same degree of loss aversion leads to similar wagering behaviour following reports of both signal and noise trials. It is possible that these criteria (*c *+ *m* and *c* − *m*) are independently specified; for example, it may be easier to be sure of seeing something than to be sure of not seeing something. To examine such effects, we split $d_{w}^{\prime }$ and *c_w_* by whether the first response had been “word” or “non-word” (equivalent to calculating separate hit and false alarm rates for the left- and right-hand sides of the distributions in Fig. 1b). These were entered into a 2 (Type I response) × 3 (visibility) × 4 (wager size) ANOVA. A robust main effect of Type I response on *C_w_* was found (*F*_(1,12)_ = 37.3, *p *< .0001), which was driven by a more conservative criterion for wagering following non-word (mean *c_w_ *= 0.60) compared to word responses (mean *c_w_ *= −0.16). Importantly, however, this dependence of *c_w_* on the initial decision was highly consistent across both wager size (interaction with wager size; *F*_(2,24)_ < 1, *p *> .9) and changes in stimulus visibility (*F*_(3,36)_ < 1, *p *> .6), indicative of a stable baseline shift that does not affect the experimental manipulations of interest. In addition, we confirmed that the effects of wager size on $d_{w}^{\prime }$ and *c_w_* (reported in Section 3.3) were not qualified by interactions with stimulus or Type I response (word/non-word; all *F* < 1).

Finally, we note that the results presented in Figs. 5b and 6 are derived from point estimates of $d_{w}^{\prime }$ and *c_w_*, which in turn rely on the assumption of equal variance Gaussian confidence distributions, *f*(*x*|*C*) and *f*(*x*|*I*) (Green & Swets, 1966). In fact, the Type II probability distributions used in the model are known to deviate from these assumptions (Fig. 1b; Galvin et al., 2003). Such deviations produce interactions between Type II *d*′ and shifts in criterion (Evans & Azzopardi, 2007). However, we consider it unlikely that our observation of decreasing $d_{w}^{\prime }$ with wager size is an artefact of a criterion shift, for two reasons. First, the prediction based on the Type II ROC curve is that for higher wagers, more conservative criteria would actually result in slight increases in $d_{w}^{\prime }$ (Fig. 5a, left-hand panel; see also Evans & Azzopardi, 2007). The opposite is seen in the data for low-visibility stimuli (Fig. 5b, left-hand panel and Fig. 6). Second, the same effects hold when an overall measure of the wagering “error” rate is calculated (overall proportion of false alarms and misses in Table 1). Collapsing over stimulus visibility, greater errors were seen in the usage of a £10 wager (45 ± 5%) than in the usage of a £1 wager (34 ± 3%).

## 4 Discussion

Measuring conscious awareness is fraught with controversy (Seth, Dienes, Cleeremans, Overgaard, & Pessoa, 2008) where even the simplest of methods, asking for subjective reports, is dogged by the conundrum of response bias (Eriksen, 1960). Recently, wagering contingent on decision performance (post-decision wagering) has been proposed as a direct and intuitive measure of conscious awareness (Koch & Preuschoff, 2007; Persaud et al., 2007; Persaud & McLeod, 2008). In the present study, we examine how economic factors affect wagers placed on perceptual decisions, and consider the theoretical implications our results have for post-decision wagering as a measure of subjective awareness.

Our signal detection model, derived from equations developed by Galvin et al. (2003), defines the transformations of noisy sensory evidence into confidence distributions on which wagering responses are based. Due to inherent loss aversion (Kahneman & Tversky, 1979; Schurger & Sher, 2008), this model predicts that advantageous wagering (Type II hits) on a perceptual event depends on the size of the gamble, despite sensory evidence remaining constant. Our psychophysics data confirm this pattern of predicted results. Our findings suggest that loss aversion is a key modulator of the linkage between perceptual sensitivity and confident wagering. This effect was true even for the highest stimulus visibility, demonstrating a labile linkage between perception and behaviour.

It is clear from these results that the typical post-decision wagering measure – advantageous wagering, or Type II hit rate – is subject to a bias induced by loss aversion, despite underlying Type I sensitivity as measured by responses to the stimulus remaining constant. Data from a related task requiring uncertainty monitoring (a scholastic aptitude test where omission of responses was allowed), revealed an analogous reduction in test score when the penalty for incorrect answers was increased (Higham, 2007). Higham went on to show that this reduction was due to more conservative Type II response criteria in the high-penalty condition. Similarly, our model predicts that the effect of loss aversion on advantageous wagering is largely due to a criterion (*c_w_*) shift, reducing the propensity of the subject to opt for the high wager regardless of whether her first response was correct or not. In contrast, Type II wagering efficiency ($d_{w}^{\prime }$; the difference between good and bad wagers in Table 1) should remain relatively constant in the face of changing wager size. In our psychophysical task, we find predictable effects of the wager size on Type II response criteria, but also observe decreases in $d_{w}^{\prime }$ as a function of wager size for near-threshold stimuli. In other words, for the same underlying task performance, participants become worse at discriminating between their own correct and incorrect responses as wager size increases.

Previous work has shown that Type II sensitivity and criterion are not in fact independent when derived from contingency tables such as Table 1 (Evans & Azzopardi, 2007), assuming equal variance Type I distributions (Galvin et al., 2003). Indeed, similar (but more subtle) increases in sensitivity as a function of wager size were predicted by our model (Fig. 5a, left panel). However, the empirical effect we observe is a *decrease* in $d_{w}^{\prime }$ for near-threshold stimuli as wager size increases (Fig. 5b, left panel and Fig. 6). What might cause this decrease in wagering efficiency on high-wager blocks? It is possible that the prospect of high wagers interferes with participants’ performance monitoring on low-visibility (difficult) trials. However, this account might also predict decreases in Type I task performance, which are not seen in our data. We note that recent evidence suggests that the prospect of large rewards (comparable to those used in the present study) causes paradoxical performance decrements (Mobbs et al., 2009; see also Baumeister, 1984). Whether such influences of large rewards on behaviour could also lead to a reduction in performance monitoring, consistent with the present data, is a question that requires further study.

### 4.1 Generation of confidence

Two inherent assumptions of our model deserve attention in order to illustrate the current limitations of a purely signal detection-based theory of confidence. A first assumption is that the initial sensory judgement is unbiased. Empirically, however, there was a consistent bias towards responding “word” for the medium and high stimulus visibility conditions. As this bias was consistent across wager size, it is unlikely to affect our conclusions regarding the effects of wager size on behaviour. However, it does affect how we characterise the link between underlying stimulus distributions and confidence, an issue we consider below. A second assumption of the model is that the criteria for wagering are symmetrical (Fig. 1b). This corresponds to equating the confidence required to wager high for words and non-words. In our behavioural data, we see a more conservative criterion for wagering high following non-word decisions. However, this shift was expressed consistently across conditions, leaving unaffected the main conclusions we infer regarding changes in *c_w_* and $d_{w}^{\prime }$ as a function of wager size. In our model, wagering criteria are symmetric because the same utility function is applied to symmetric signal and noise distributions. Within this framework, a baseline difference in wagering criteria may be caused by the variance of the signal (word) distribution being greater than the noise (non-word) distribution, leading to a more prominent *f*(*I*|*x*) distribution following non-word responses and requiring more conservative criteria to maintain a given level of wagering performance. Interestingly, greater signal variance when compared to noise in empirical ROCs has been documented in the memory literature (Ratcliff, Sheu, & Gronlund, 1992). However, it is unclear from the present data whether distributional or response factors are the main drivers of the observed asymmetry in wagering criteria.

These behavioural deviations from the model hint at a deeper issue regarding the relationship between Type I task performance and metacognitive confidence (Baranski & Petrusic, 2001; Busey, Tunnicliff, Loftus, & Loftus, 2000; Higham et al., 2009). In our signal detection model, probability distributions over the stimulus are assumed to deterministically give rise to Type II confidence distributions, without invoking the notion of intermediate processing stages. Higham et al. (2009) identify this generation of confidence from stimulus distributions as the “direct translation hypothesis”. They empirically tested this hypothesis by varying Type I response criteria in a memory paradigm, noting that Type II sensitivity should be systematically affected if Type I distributions are indeed directly translated into Type II distributions, a conclusion supported by their data (Higham et al., 2009; Experiment 3). However, recent human psychophysical data reveal dissociations between objective performance and subjective confidence, suggesting a more complex relationship between Type I and Type II distributions (Wilimzig, Tsuchiya, Fahle, Einhauser, & Koch, 2008; see also Busey & Arici, 2009). In addition, reaction time measures suggest that confidence is at least partly determined by additional processing following the decision itself (Baranski & Petrusic, 1998, 2001). In the present study, word–non-word discrimination may be a suboptimal testbed for the direct translation hypothesis, given that the evidence dimension is unlikely to be unitary. For example, some words may be easier to process than others, engendering higher fluency and thus influencing metacognitive assessment (Kelley & Lindsay, 1993; Koriat, 1993; Winkielman, Halberstadt, Fazendeiro, & Catty, 2006). Future work is needed examine the empirical form of Type I and Type II distributions under simpler psychophysical conditions.

### 4.2 Post-decision wagering and awareness

What do the present results say about post-decision wagering as a measure of awareness? On a psychological level, the use of advantageous wagering counts (Type II hit rate) is confounded by factors (presumably) external to stimulus awareness (see also Dienes & Seth, 2009). For example, blindsight subject GY was seen to wager high 48% of the time after correct responses to stimuli in his blind hemifield (Persaud et al., 2007; p. 257). Persaud and colleagues argue that as this proportion is no better than chance (50%), it is evidence for a lack of awareness. However, it is clear from the model and data we present here that simply altering the size of the wager can manipulate this proportion to be consistent with higher or lower awareness. Application of Type II SDT measures to GYs response counts might be a more convincing demonstration of a lack of awareness (Persaud et al. (2007), their Supplementary Table 6; but see Clifford et al. (2008)). Again, however, this measure is not perfect: we find changes in wager size produced unpredictable effects on $d_{w}^{\prime }$ for the types of near-threshold stimuli often used in consciousness research, and particular forms of the payoff matrix can produce values of $d_{w}^{\prime }$ consistent with a lack of awareness (Clifford et al., 2008). Further problems arise when making comparisons across subjects, or when comparing patients with controls: in our model fit, loss aversion varied considerably between subjects (and may vary in an even more unpredictable fashion within different patient populations), leading to the interpretation of wagering responses being confounded by individual differences in the subjective utility of the gamble (Dienes & Seth, 2009; Schurger & Sher, 2008). There are obvious instances where the ease of use and nonverbal nature of wagering may outweigh such drawbacks, for instance when measuring awareness in non-human animals and children. In these cases, care should be taken to address potentially illusory changes in awareness caused by economic factors.

On a philosophical level, even if it is possible to control for the confounding effects of loss aversion, it is unclear whether awareness can be inferred from successful wagering (Seth, 2008). Recent data demonstrates that adaptive, value-based responses can be made in response to stimuli that are below an objective threshold of awareness (Pessiglione et al., 2007, 2008). Indeed, from an economist’s perspective, a post-decision wager is a gamble in which the sensory uncertainty determines the probabilities of winning and losing. Graded changes in either the stimulus or outcome utilities will produce probabilistic changes in wagering behaviour, which may not have obvious mappings to particular states of consciousness. In contrast, explicitly taking into account this graded nature of processing using, for example, direct confidence rating scales may offer more robust means of assessing both objective sensitivity and subjective meta-sensitivity (Szczepanowski & Pessoa, 2007). In this regard our results emphasise the importance of considering both stimulus and response variables when assessing conscious awareness (Clifford et al., 2008; Evans & Azzopardi, 2007; Hulme, Friston, & Zeki, 2009).

In summary, using a combination of signal detection theory and psychophysics we show that the translation of perceptual sensitivity into a post-decision wagering response is systematically affected by economic variables, in this instance by loss aversion. Type II signal detection measures reveal that changes in behaviour induced by changes in wager size are largely driven by changes in criterion, consistent with our model. However, when stimulus visibility is low and wagers are large, a reduction in wagering efficiency is also seen. Indeed, the complex interaction between objective stimulus visibility, wager size and the subsequent willingness to gamble casts doubt on an assertion (Persaud et al., 2007) that post-decision wagering is a direct index of subjective awareness, despite its intuitive nature. Such interactions raise intriguing questions for future work into the relationship between stimulus processing, subjective awareness and the generation of metacognitive confidence.

## Figures and Tables

**Table 1:** Categorisation of subjects’ wagering responses for a Type II signal detection analysis.

Type I decision	High wager	Low wager
Correct	Hit	Miss
Incorrect	False alarm	Correct rejection
