# Supplementary material for: Effects of loss aversion on post-decision wagering: Implications for measures of awareness
Source: Conscious Cogn. 2010 Mar;19(1):352–63. doi: 10.1016/j.concog.2009.11.002 (PMC2842936; doi:10.1016/j.concog.2009.11.002)
Supplement: Supplementary Material [file mmc1.doc]

**Effects of loss aversion on post-decision wagering: Implications for measures of awareness**

**Stephen M. Fleming & Raymond J. Dolan**

**SUPPLEMENTARY MATERIAL**

**SUPPLEMENTARY METHODS**

Utility curves specify the transformation of absolute value into a subjective utility. Prospect Theory (Kahneman & Tversky, 1979) proposes the following features of utility curves: decreasing marginal utility, indexed by curves being convex in gains and concave in losses, and loss aversion, leading to the curve for losses being steeper than the curve for gains.

We fitted curves of this type to our post-decision wagering data. Three models were considered: symmetric, power, and loss averse + power. Each successive model has one additional parameter, and exemplar forms of each curve are shown below:

**Symmetric**

**Power**

**Power + loss averse**

Each curve is governed by the general equations:

$$U=V^{r},V>0$$

$$U=s(-V)^{t},V<0$$

In model 1 (symmetric), s is a free parameter and r = t. In model 2 (power), s = 1, and r and t are free parameters. In model 3, s, r and t are free parameters.

Asymmetric utility curves predict that the likelihood of wagering high (*β*_w_, Eq. 5 of main paper) should shift as a function of wager size. However, this is only true if the asymmetry is itself a function of value. This is only the case for models 2 and 3, in which r ≠ t, as shown in the panel below. As such, model 1 was unable to predict changes in wagering behaviour as a function of wager size (despite including a loss aversion coefficient, *s*), due to a flat parameter space. The fit of this model was correspondingly very poor (BIC = 71510, compared to BIC = 14419 for the next best model 2), and we did not consider it further. However, it is interesting to note that while the primary aim of this study was to investigate the effects of utility curves on signal detection parameters, converse inferences can be made. Given our behavioural findings that changes in wager size altered the criterion for wagering for a fixed stimulus visibility, the utility curve for mixed gambles must incorporate nonlinearities in the relationship of the gain to the loss domain. This is parameterised here as a difference in the gain (r) and loss (t) powers (see also Fennema & Van Assen, 1999; Kobberling & Wakker, 2005).


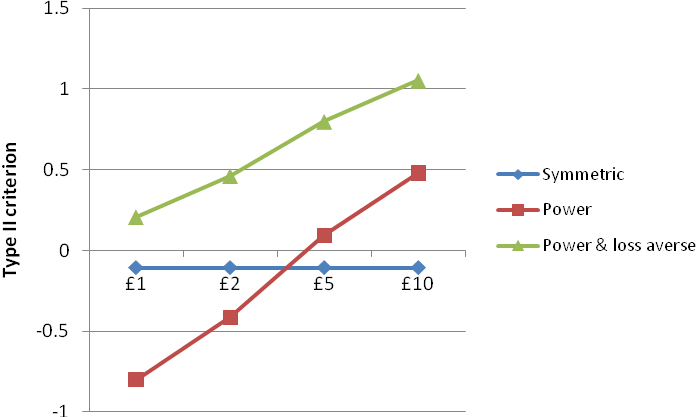


As reported in the main text, the fit of the loss averse + power model was significantly better than the power model, despite the penalty imposed by the BIC for additional parameters. The mean loss aversion coefficient of 2.74 leads to a wager of £1 being translated into utilities of £1 gain and £2.74 loss. However, loss aversion *increases* as the wager size increases, such that wager of £10 is treated subjectively as a potential £2.95 gain or a potential £18.83 loss. This relationship between positive and negative utility is tabulated below for the four sizes of wager.

| **Wager** | **U(x), Model 3** | **-U(x), Model 3** |
| --- | --- | --- |
| £1 | £1 | -£2.74 |
| £2 | £1.39 | -£4.89 |
| £5 | £2.13 | -£10.54 |
| £10 | £2.95 | -£18.83 |

**SUPPLEMENTARY REFERENCES**

Fennema, H. & Van Assen, M. (1999). Measuring the utility of losses by means of the tradeoff method. *Journal of Risk and Uncertainty, 17,* 277-295.

Kahneman, D. & Tversky, A. (1979). Prospect Theory - Analysis of decision under risk. *Econometrica, 47,* 263-291.

Kobberling, V. & Wakker, P. P. (2005). An index of loss aversion. *Journal of Economic Theory, 122,* 119-131.
